# Lassa Fever in Post-Conflict Sierra Leone

**DOI:** 10.1371/journal.pntd.0002748

**Published:** 2014-03-20

**Authors:** Jeffrey G. Shaffer, Donald S. Grant, John S. Schieffelin, Matt L. Boisen, Augustine Goba, Jessica N. Hartnett, Danielle C. Levy, Rachael E. Yenni, Lina M. Moses, Mohammed Fullah, Mambo Momoh, Mbalu Fonnie, Richard Fonnie, Lansana Kanneh, Veronica J. Koroma, Kandeh Kargbo, Darin Ottomassathien, Ivana J. Muncy, Abigail B. Jones, Megan M. Illick, Peter C. Kulakosky, Allyson M. Haislip, Christopher M. Bishop, Deborah H. Elliot, Bethany L. Brown, Hu Zhu, Kathryn M. Hastie, Kristian G. Andersen, Stephen K. Gire, Shervin Tabrizi, Ridhi Tariyal, Mathew Stremlau, Alex Matschiner, Darryl B. Sampey, Jennifer S. Spence, Robert W. Cross, Joan B. Geisbert, Onikepe A. Folarin, Christian T. Happi, Kelly R. Pitts, F. Jon Geske, Thomas W. Geisbert, Erica Ollmann Saphire, James E. Robinson, Russell B. Wilson, Pardis C. Sabeti, Lee A. Henderson, S. Humarr Khan, Daniel G. Bausch, Luis M. Branco, Robert F. Garry, the Viral Hemorrhagic Fever Consortium

**Affiliations:** 1 Department of Biostatistics and Bioinformatics, Tulane School of Public Health and Tropical Medicine, New Orleans, Louisiana, United States of America; 2 Lassa Fever Program, Kenema Government Hospital, Kenema, Sierra Leone; 3 Sections of Infectious Disease, Departments of Pediatrics and Internal Medicine, School of Medicine, Tulane University, New Orleans, Louisiana, United States of America; 4 Department of Microbiology and Immunology, Tulane University, New Orleans, Louisiana, United States of America; 5 Corgenix, Inc., Broomfield, Colorado, United States of America; 6 Department of Tropical Medicine, Tulane School of Public Health and Tropical Medicine, New Orleans, Louisiana, United States of America; 7 Biofactura Inc., Rockville, Maryland, United States of America; 8 Autoimmune Technologies, LLC, New Orleans, Louisiana, United States of America; 9 Vybion Inc., Ithaca, New York, United States of America; 10 Department of Immunology and Microbial Science, The Scripps Research Institute, La Jolla, California, United States of America; 11 FAS Center for Systems Biology, Department of Organismic and Evolutionary Biology, Harvard University, Cambridge, Massachusetts, United States of America; 12 Broad Institute, Cambridge, Massachusetts, United States of America; 13 Department of Microbiology and Immunology, University of Texas Medical Branch at Galveston, Galveston, Texas, United States of America; 14 Department of Biological Sciences, College of Natural Sciences, Redeemers University, Redemption City, Ogun State, Nigeria; 15 The Skaggs Institute for Chemical Biology, The Scripps Research Institute, La Jolla, California, United States of America; 16 Department of Immunology and Infectious Disease, Harvard School of Public Health, Boston, Massachusetts, United States of America; 17 Zalgen Labs, LLC, Germantown, Maryland, United States of America; Centers for Disease Control and Prevention, United States of America

## Abstract

**Background:**

Lassa fever (LF), an often-fatal hemorrhagic disease caused by Lassa virus (LASV), is a major public health threat in West Africa. When the violent civil conflict in Sierra Leone (1991 to 2002) ended, an international consortium assisted in restoration of the LF program at Kenema Government Hospital (KGH) in an area with the world's highest incidence of the disease.

**Methodology/Principal Findings:**

Clinical and laboratory records of patients presenting to the KGH Lassa Ward in the post-conflict period were organized electronically. Recombinant antigen-based LF immunoassays were used to assess LASV antigenemia and LASV-specific antibodies in patients who met criteria for suspected LF. KGH has been reestablished as a center for LF treatment and research, with over 500 suspected cases now presenting yearly. Higher case fatality rates (CFRs) in LF patients were observed compared to studies conducted prior to the civil conflict. Different criteria for defining LF stages and differences in sensitivity of assays likely account for these differences. The highest incidence of LF in Sierra Leone was observed during the dry season. LF cases were observed in ten of Sierra Leone's thirteen districts, with numerous cases from outside the traditional endemic zone. Deaths in patients presenting with LASV antigenemia were skewed towards individuals less than 29 years of age. Women self-reporting as pregnant were significantly overrepresented among LASV antigenemic patients. The CFR of ribavirin-treated patients presenting early in acute infection was lower than in untreated subjects.

**Conclusions/Significance:**

Lassa fever remains a major public health threat in Sierra Leone. Outreach activities should expand because LF may be more widespread in Sierra Leone than previously recognized. Enhanced case finding to ensure rapid diagnosis and treatment is imperative to reduce mortality. Even with ribavirin treatment, there was a high rate of fatalities underscoring the need to develop more effective and/or supplemental treatments for LF.

## Author Summary

Lassa fever (LF) is a major public health threat in West Africa. After the violent civil conflict in Sierra Leone (1991 to 2002) ended, the LF research program at Kenema Government Hospital (KGH) was reestablished. Higher CFRs in LF patients were observed compared to studies conducted prior to the civil conflict. The criteria used for defining the stages of LF and differences in sensitivity of the assays used likely account for these differences. LF may be more widespread in Sierra Leone than recognized previously. Peak presentation of LF cases occurs in the dry season, which is consistent with previous studies. Our studies also confirmed reports conducted prior to the civil conflict that indicate that infants, children, young adults, and pregnant women are disproportionately impacted by LF. High fatality rates were observed among both ribavirin treated and untreated patients, which underscores then need for better LF treatments.

## Introduction

Viral hemorrhagic fevers are among the most feared diseases due to their high case fatality rates (CFRs), severe clinical presentations and ease of transmission. Unlike most viral hemorrhagic fevers, which are recognized only when outbreaks occur, Lassa fever (LF) is endemic in West Africa, with an estimated tens of thousands of cases annually [1]. LF was first recognized in 1969 following the deaths of two missionary nurses during an outbreak in northeastern Nigeria [2]–[4]. The populations of Sierra Leone, Guinea, Liberia and other West African countries were subsequently also shown to be at risk for LF [5]–[8]. Humans become infected with LASV by exposure to the excreta of its reservoir *Mastomys natalensis*, also known as the “multimammate rat” [9], [10]. As in the first reported cases, secondary human-to-human transmission of LASV also occurs through direct contact with infected blood or bodily secretions. Deaths among nurses, doctors and other healthcare workers occasionally occur when adherence to barrier nursing and contact precautions are not maintained [11].

After an incubation period of 5–16 days, LF typically begins with fever and a host of other non-specific manifestations, including headache, sore throat, myalgia, and gastrointestinal symptoms [5], [12]–[16]. More specific manifestations such as conjunctival injection, retrosternal pain, facial swelling, and mucosal and gastrointestinal bleeding occur in less than a third of cases. The non-specific clinical presentation makes LF extremely difficult to recognize on clinical grounds alone, especially in the early phases. Prompt laboratory diagnosis is therefore essential. Unfortunately, laboratory diagnosis has generally not been available in the endemic areas, impeded by the unavailability of diagnostic reagents, which have traditionally been based on inactivated cultures of LASV, a process that can only safely be performed in the very few high containment laboratories around the world. Death from LF is attributed to diminished effective circulating volume, shock, and multi-organ system failure [17]. The antiviral drug ribavirin can be effective, but only when given early in the course of disease, ideally within the first six days [18]. There is no approved LF vaccine.

The Eastern Province of Sierra Leone is considered to have the world's highest LF incidence [6]. In 1976, the Sierra Leone Ministry of Health and Sanitation (MoHS) with support from the United States Centers for Disease Control and Prevention (CDC) established LF treatment wards and a diagnostic laboratory in the Eastern Province [19]. Kenema Government Hospital (KGH) was an important site for LF clinical and laboratory research throughout the remainder of the 1970s and the 1980s [12], [13], [20]. The violent civil conflict from 1991 to 2002, sometimes referred to as the Blood Diamonds War, forced suspension of CDC involvement in LF research at KGH in 1993. Following the cessation of hostilities, a consortium of LF researchers in close collaboration with the MoHS began rebuilding the scientific infrastructure at KGH, with the development of improved laboratory diagnosis for LF as a major focus [19], [21]–[24]. Here, we summarize the demographics and epidemiology of patients presenting to KGH with suspected LF in the decade following the end of the civil conflict.

## Methods

### Ethics statement

The Tulane University Institutional Review Board and the Sierra Leone Ethics Committee approved this project. Patients were referred to the KGH Lassa Ward from regional health centers or the hospital's general ward on the basis of suspicion of LF. Patients were cared for by the ward's trained staff [19]. All adult subjects provided written informed consent to analyze and publish laboratory and clinical data. A parent or guardian of any child participant provided written informed consent on their behalf.

### Patient selection and data collection

To be considered for admission to the KGH Lassa Ward, subjects were required to satisfy the LF case definition criteria (Table 1) according to an evaluation questionnaire. In many cases, we only received blood samples from referral centers, and survival outcome results were not always known. The sample population for this work was based on the availability of Ag and IgM ELISA test results, regardless of whether these patients presented to KGH. Since screening based on anti-LASV IgG-capture ELISA was not routinely conducted at the KGH until 2011, it was not considered an inclusion criterion. Observations were excluded if they were for subjects residing outside of Sierra Leone (the KGH Lassa Laboratory routinely receives samples from Liberia for testing, and occasionally receives samples from Guinea) or if they were classified as contacts of an index LF case. We imposed several additional criteria in an effort to exclude invalid test results. Specifically, we required that raw laboratory data be available for both the Ag and IgM ELISA tests. Also, raw laboratory data was considered valid only if each of its OD values was non-negative (for the patient sample and its positive and negative controls) and the OD values for the positive controls were greater than those for its corresponding negative control. These restrictions resulted in the n = 1740 observations used for the study (Table S1). These samples were used to generate the final test results using a cutoff-based approach. The test result cutoffs were calculated by year of presentation to control for the environmental changes and temporal improvements at the KGH laboratory. These results were then linked to all of the clinical, epidemiological, and treatment data available. The study was not designed as a longitudinal study, but we did observe n = 362 serial samples to address questions involving LF seroconversions or changes in Ag or antibody levels.

**Table 1:** Case definition for suspected cases of LF.

• Known exposure to a person suspected to have Lassa fever	
• Fever >38°C for less than three weeks PLUS	
• Absence of signs of local inflammation AND	
• Two major signs or one major and two minor signs	
**Major Signs**	**Minor Signs**
• Bleeding	• Headache
• Swollen neck or face	• Sore throat
• Conjunctivitis or sub-conjunctival hemorrhage	• Vomiting
• Spontaneous abortion	• Diffuse abdominal pain/tenderness
• Petechial or hemorrhagic rash	• Chest/retrosternal pain
• New onset of tinnitus or altered hearing	• Cough
• Persistent hypotension	• Diarrhea
• Absence of clinical response after 48 hrs to anti-malarial and/or broad spectrum antibiotic therapy	• Generalized myalgia or arthralgia
	• Profuse weakness

Modified from Khan et al., 2008 [19].

All demographic, clinical, and treatment data were entered into a Microsoft Access database using electronic questionnaire forms. Raw laboratory data was maintained using Excel spreadsheets with standard structures. The data were progressively cleaned and curated via regular on-and off-site data audits, where data were checked against their questionnaire forms and laboratory notebooks. Raw laboratory data were compiled using a suite of Microsoft Excel macros. All available data sources were linked using the SAS System (version 9.3; SAS Institute, Cary, NC.

### LF recombinant antigen immunoassays at KGH

We have focused on the development of recombinant antigen-based immunoassays for diagnosis of LF at KGH. These assays have the safety advantages of not requiring BSL-4 cell culture for production [22], [24]. Performance of the Recombinant Lassa (ReLASV) Diagnostics system versus detection of LASV viremia by reverse transcriptase polymerase chain (RT-PCR) was evaluated in a clinical study conducted at KGH in 2012–13 (unpublished data). Criteria for inclusion in the clinical study were based on the suspected LF case definition (Table 1). Limits of detection and quantitation of the recombinant antigen-based LF enzyme-linked immunosorbent assays (ELISA) used were based on the upper 95th percentile obtained with a panel of sera from U.S. and Sierra Leonean donors lacking detectable LASV antigens or immunoglobulin M or G (IgM, and IgG) antibodies to LASV recombinant proteins. IgG depletion studies (protein A) demonstrated that the LASV IgM assay is only detecting anti-LASV IgM, not IgG and conversely that the IgG assay has a high specificity for anti-LASV IgG. Upon presentation to the LF clinical study program, screening with the ReLASV diagnostic assays, upon presentation to the LF clinical study program, was capable of identifying 95% of active LF cases as confirmed by RT-PCR performed at KGH, rising IgM titers or IgM to IgG seroconversion (Table S2) (unpublished data). The diagnostic assays being utilized miss a low percentage (<5%) of resolving or less severe cases with low virus load that are generally associated with low mortality. Combining the NP antigen detection (Ag-capture ELISA) results with anti-LASV IgM reactivity (IgM-capture ELISA) generated a 98% negative predictive value, which further demonstrates its capability as an on-site point of care screening protocol. A lateral flow immunoassay (rapid diagnostic test, RDT) requiring only a drop of blood (30 µl) obtained with a safety lancet and capable of detecting LASV antigenemia within 15 minutes was also developed.

Sequence variability of LASV challenges both immune-based and molecular assays [25]. The immunoassays used in the current study are designed to detect antigens of LASV circulating in Sierra Leone or surrounding countries or antibodies to these viruses. We used high-throughput sequencing to generate a catalogue of LASV genomic data (unpublished data). We confirmed prior studies indicating that there are three diverse LASV lineages in Nigeria (I–III), but only a single lineage (IV) in Sierra Leone. We have detected only lineage IV LASV in Sierra Leone in these genomic screens. The Ag-capture ELISA we have developed using LASV Josiah (lineage IV) recombinant proteins and antibodies to these proteins show reactivity to BSL-4 grown LASV Josiah, but reduced sensitivity to LASV of Nigerian lineages II and III. The ReLASV immunoassays failed to react with New World arenavirus antigens (Junin or Pichinde viruses) or several RNA or DNA virus antigens (dengue virus type 1, 2, 3, 4, influenza A virus, vesicular stomatitis virus, respiratory syncytial virus, cytomegalovirus, poxvirus and others).

### Statistical analysis

Data were analyzed using the SAS System (version 9.3; SAS Institute, Inc., Cary, NC). Hypotheses involving dichotomous response variables were tested using Fisher's exact test or logistic regression. For logistic regression models with multiple independent predictors, all pairwise interaction terms were included in the model. Count outcomes were modeled using Poisson regression, and distributional comparisons were carried out using the Kolmogorov-Smirnov approach. Gaussian kernel smoothing was used to smooth the categorical serostatus age distributions. Analyses were two-tailed with a significance threshold set at p<.05

### Accession number

Recombinant nucleoprotein, glycoprotein and Z protein from lineage IV Lassa virus Josiah strain (ADY11071) were use used in the IgM and IgG-capture ELISAs. These recombinant proteins were also used to immunize goats and mice for use in the ReLASV Ag-capture ELISA and RDT.

## Results

### Reestablishment of KGH as a treatment and research center for LF

Admission to the KGH Lassa Ward on the basis of clinical suspicion of LF continued throughout the extremely difficult years of the civil conflict, with up to 500 suspected cases in some years [1], [19], [26]. Paradoxically, after cessation of hostilities, and with the MoHS facing an enormous rebuilding challenge and the departure of many of the humanitarian assistance groups previously confronting LF, there was a period where few resources were available for patients suffering from LF; from 2004 to 2008 there were 405 confirmed or probable LF cases presenting to KGH, with a peak of 227 in 2004. Most were diagnosed only on clinical grounds or occasionally using nonrecombinant antigen-capture ELISA [27].

**Figure 1:**
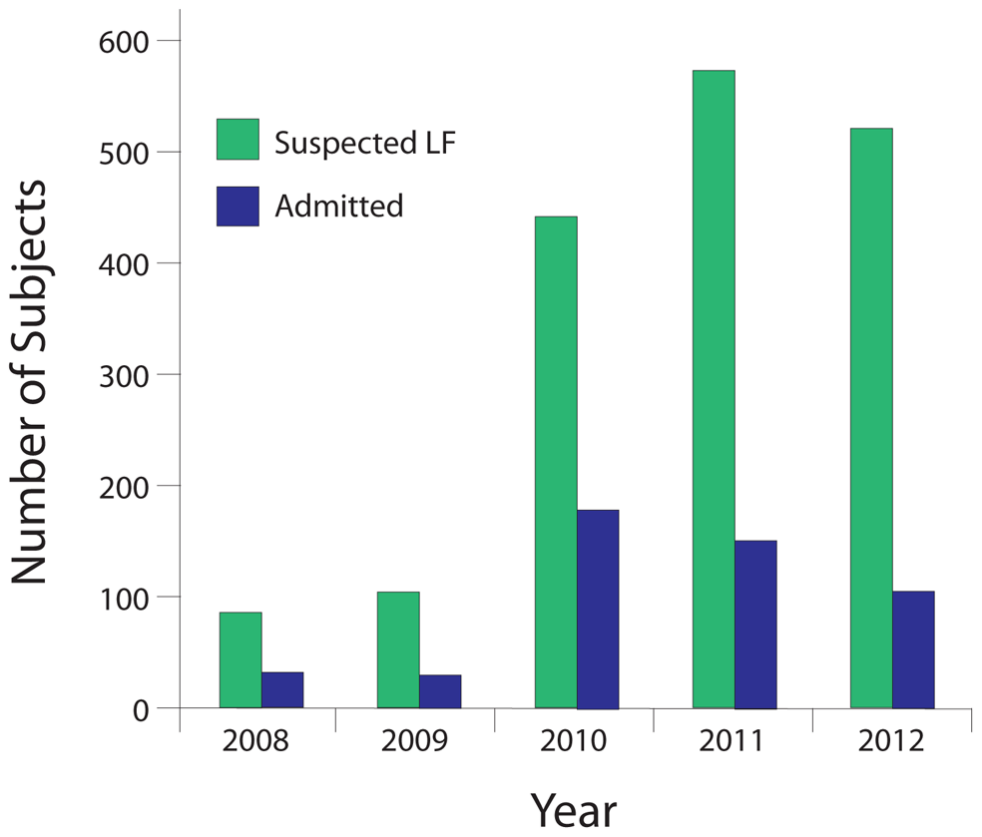
Suspected cases of LF evaluated at the KGH Lassa Laboratory and numbers of patients admitted to the KGH Lassa Ward, 2008–12. Non-admitted patients include those where only blood samples were submitted for screening from referral health-posts, patients dying en route to the hospital (DOA = dead on arrival), and patients not meeting the LF suspected case criteria (Table 1). Characteristics of study patients are compiled in Table S1.

Our group and others participated in efforts to restore and enhance both clinical and laboratory-based research on LF at KGH [19]. Recombinant antigen-based ELISAs were developed and implemented and have been used consistently at the KGH Lassa Ward since 2008, with the addition of the lateral flow immunoassay in 2010. The number of suspected LF cases presenting to KGH dramatically increased from 85 in 2008 to 529 in 2012, with a peak of 575 in 2011 (Fig. 1). The number of patients admitted to the KGH Lassa Ward peaked at 164 in 2010, then decreased to 109 in 2012 (Fig. 1). The reduction in admitted patients after 2010 corresponds to the introduction of the LF lateral flow immunoassay, which permits avoidance of some unnecessary admissions to the Lassa ward.

A positive result on the LASV antigen-capture ELISA or lateral flow immunoassay indicates that the subject is viremic and confirms the clinical diagnosis of LF. Among patients meeting the case definition of LF (Table 1) who were evaluated at the KGH Lassa Ward from 2008–2012, 11% (190/1740) were antigenemic on the first blood draw. These patients were also evaluated for the presence of anti-LASV IgM: 8% (140/1740) were Ag+/IgM−, while 3% (50/1740) were Ag+/IgM+. Among non-antigenemic patients, 23% (407/1740) have serum IgM antibodies to LASV. As discussed below, the presence of anti-LASV IgM antibodies can represent several stages in LF or a prior symptomatic or asymptomatic LASV infection. Diagnosis of recent LF in admitted subjects without antigenemia is revealed in some cases by additional blood draws to determine if the subject demonstrates an increasing anti-LASV IgM titer or shows a class switch to anti-LASV IgG. Of the suspected LF patients 65% (1143/1740) were Ag−/IgM− and therefore considered to have a non-Lassa febrile illness (NLFI).

### Case fatality rates by serostatus

**Figure 2:**
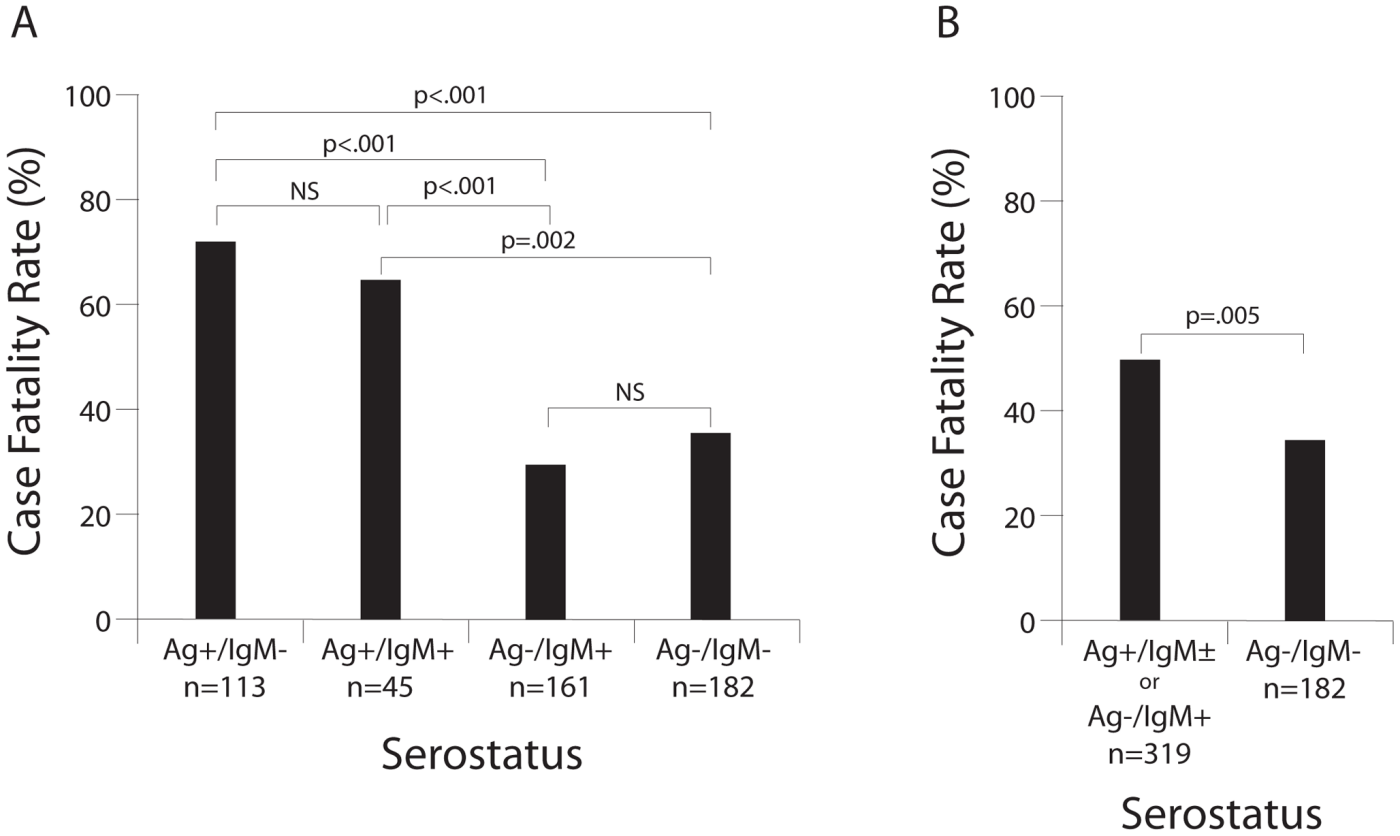
CFRs in suspected LF cases presenting to the KGH Lassa Ward by serostatus, 2008–12. Panel A: CFR by serostatus. The presence of LASV Ag and anti-LASV IgM in serum of patients with verifiable outcomes was assessed by recombinant Ag− and IgM− capture ELISA, respectively. Panel B: Alternative calculation of CFRs. Ag+/IgM± plus Ag−/IgM+ compared to Ag−/IgM−. Statistical significance was determined using a logistic regression model predicting CFR (Table S3). NS = not significant.

The overall CFR in antigenemic LF cases (Ag+/IgM±) with verifiable outcomes was 69% (109/158). The CFR was similar in Ag+ cases regardless of their anti-LASV IgM result (62% (28/45) for Ag+/IgM− and 72% (81/113) for Ag+/IgM+ cases; Fig. 2A). In contrast, the CFR in Ag−/IgM+ patients (first blood draw) was 29% (46/161), which was not statistically different from the NLFI patients (36% [65/182]; Fig. 2A, Table S1). All subjects included in the study met the case definition for LF. The patients were generally severely ill, irrespective of whether they were diagnosed with LF, which likely accounts for the high CFR in the Ag−/IgM+ and Ag−/IgM− groups. Nearly all patients were unresponsive to anti-malaria and antibiotic therapy, and remained ill. Despite anti-malaria treatment, we found that more than half of a sample of NLFI subjects analyzed had detectable levels of *Plasmodium falciparum* (data not shown). HIV testing was not performed. Studies of NLFIs in this population are of interest, but an extensive characterization was not possible due to resource and infrastructure constraints during this project period. It should be noted that CFRs are based on patients with verifiable outcomes from medical records. The CFRs in the Ag− serogroup patients, who were admitted to the Lassa Ward, are almost certainly inflated relative to patients in these serogroups who were not admitted.

While Ag-positive subjects at the regional health posts were usually transferred to KGH, some did not physically present to KGH and their survival outcomes could not be determined. A few Ag+ cases refused admission or informed consent, and 32 Ag+ subjects without outcome data represented referred samples or those that refused admission or informed consent (Table S1). Including these 32 Ag+ subjects (and assuming they survived LASV infection) with the 158 Ag+ patients with verifiable outcomes results in a CFR of 57% (109/190). This lower CFR requires the caveat that verifiable outcome data was not obtained for non-admitted subjects, and it is unlikely that all survived.

In most viral infections antiviral IgM arises early after infection, and then is replaced by antiviral IgG. Prior studies have included IgM+ subjects as acute LF cases [27], [28]. Including Ag−/IgM+ with Ag+/IgM± patients results in a CFR of 49% (155/319, Fig. 2B, Tables S1, S3). Ag− patients that demonstrated increasing levels of IgM suggest the possibility of a resolving infection (post-acute LF) or a stage of early convalescence. Over 84% (70/83) of Ag−/IgM+ patients for whom more than one IgM test result was available did not show a significant increase in anti-LASV IgM levels during their stay in the Lassa Ward, suggesting that the illness for which they presented was not LF. Only one (1/13, 8%) fatality was noted among Ag−/IgM+ patients with rising anti-LASV IgM levels. These patients with rising IgM levels (post-acute or early convalescence) generally had only moderately elevated aspartate aminotransferase levels (data not shown), which is an indicator of severity in LF.

**Figure 3:**
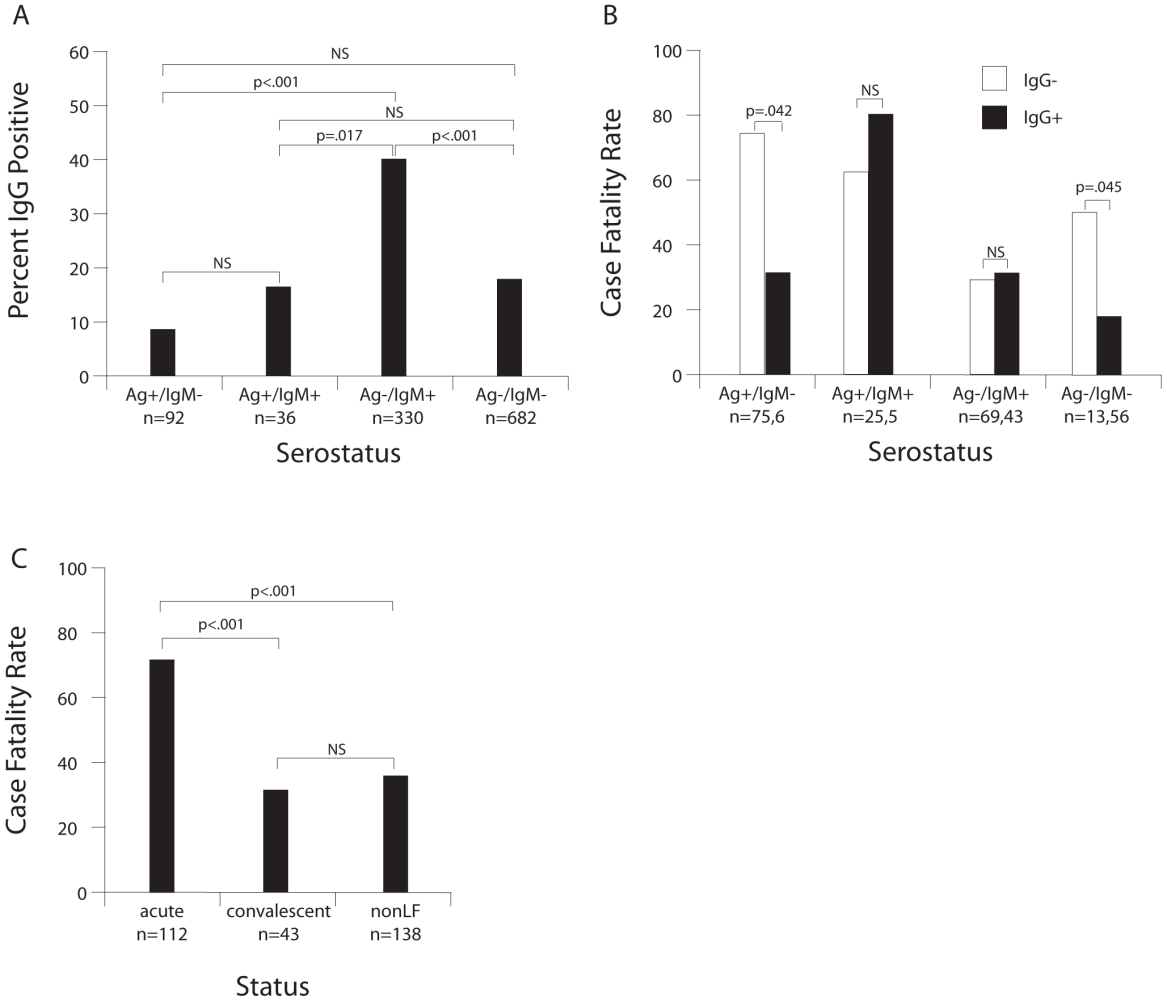
Anti-LASV IgG in suspected LF patients presenting to the Kenema Government Hospital, 2011–12. Routine anti-LASV IgG serological testing was implemented at KGH in 2011. Panel A: Percentage of patients with anti-LASV IgG by serostatus. Panel B: Case fatality rates in patients with verifiable outcomes by LASV antigen, anti-LASV IgM, and anti-LASV IgG serostatus. Panel C: Case fatality rates in patients with verifiable outcomes by an alternative assessment of LF status (acute = Ag+/IgM±/IgG±, convalescent = Ag−/IgM+/IgG+, nonLF = Ag−/IgM+/IgG− or Ag−/IgM−/IgG± LASV antigen, anti-LASV IgM, and anti-LASV IgG serostatus. Logistic regression models predicting IgG-positivity and CFRs were used to carry out within and between group comparisons (Table S4). NS = not significant.

IgG-capture ELISA was implemented for routine diagnostic testing of suspected LF patients in 2011. The prevalence of anti-LASV IgG was significantly higher in Ag−/IgM+ patients, than in Ag−/IgM− patients and patients in other serogroups (Fig. 3A). Among subjects presenting with a NLFI, as determined by the absence of LASV Ag or anti-LASV IgM, 18% (125/688 had anti-LASV IgG. CFRs of patients in the Ag+/IgM+ or Ag−/IgM+ serogroups did not differ significantly by the presence or absence of anti-LASV IgG on the first blood draw. In contrast, the CFR in patients with Ag+/IgM−/IgG+ was significantly lower than in Ag+/IgM−/IgG− patients with acute LF (p = .042, Fig. 3B, Table S4). Only six patients (two fatal) showed an Ag+/IgM−/IgG+ serological profile. This profile is consistent with a secondary LASV infection as suggested previously using indirect immunofluorescent assays [28]. Further studies of larger numbers of subjects are required prior to concluding that secondary LASV infection can occur. The CFR in NLFI patients with Ag−/IgM−/IgG+ was significantly lower than in Ag−/IgM−/IgG− patients (p = .045).

The CFR for the subset of Ag+ subjects with an IgG test result (+ or −) was 71% (79/112, Fig. 3C). Ag− patients presenting with both anti-LASV IgM+ and IgG+ may be in the convalescence stage of LF. The 33% (14/43) CFR of Ag−/IgM+/IgG+ patients was significantly lower (p<.001) than the CFR of Ag+ patients (Table S4). Most subjects presenting with Ag−/IgM+/IgG− or Ag−/IgM−/IgG± serostatus are unlikely to have presented with LF. The 37% (51/138) CFR of these subjects (non-LF) was significantly lower (p<.001) than Ag+ subjects. Ag−/IgM+/IgG− patients that seroconvert to anti-LASV IgG+ (class-switching) may also be considered to be in early LF convalescence. Out of 60 Ag−/IgM+ patients with IgG testing, a follow-up sample and verified outcome, only nine patients were IgG− on the first sample, but IgG+ on the second indicating class-switching (data not shown). None of these patients died.

### A broad geographic distribution of LF cases in Sierra Leone

**Figure 4:**
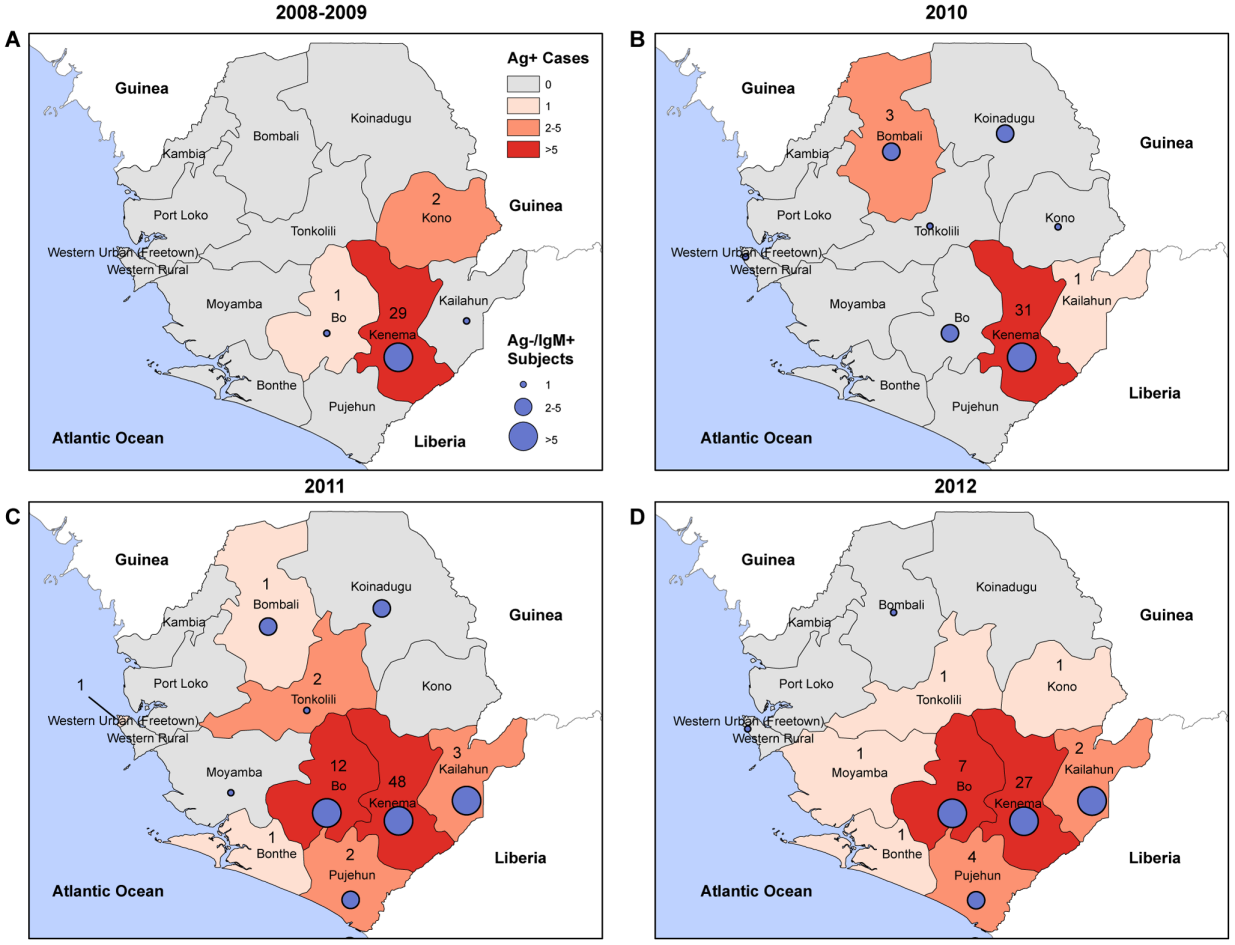
Geographic distribution of patients presenting to the KGH with LASV antigenemia and anti-LASV IgM serpositivity, 2008–12. Confirmed cases of LF as assessed by LASV Ag in serum or cases anti-LASV IgM are shown by year of presentation, district of residence and frequency of cases. Panel A: Patients presenting in 2008–9. Panel B: Patients presenting in 2010. Panel C: Patients presenting in 2011. Panel D: Patients presenting in 2012.

The majority of suspected LF cases evaluated at the KGH Lassa Laboratory from 2008–12 were residents of Kenema District in the Eastern Province of Sierra Leone, where KGH is located (Fig. 4). Cases with LF presenting to KGH from outside the recognized endemic zone were recorded in increasing frequency throughout the post-conflict period [22]. Ten of Sierra Leone's thirteen districts had at least one laboratory-confirmed LF case with antigenemia (Ag+/IgM±) from 2008–2012. IgM seropositivity, which indicates exposure to LASV, was also observed in ten of Sierra Leones thirteen districts.

### Distinct patterns of seasonal variation among suspected LF cases with different serostatus

**Figure 5:**
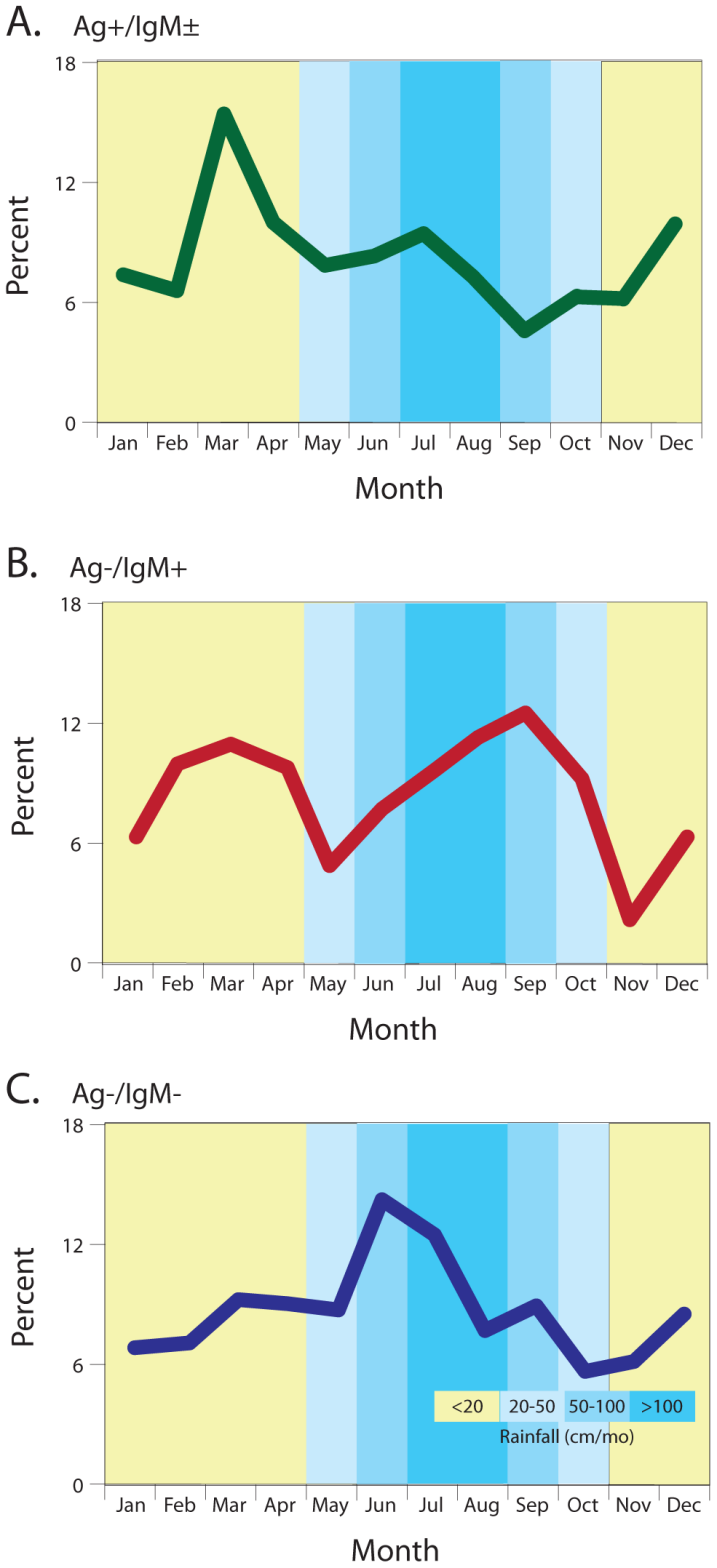
Monthly distribution of suspected LF cases presenting to the KGH Lassa Ward by serostatus, 2008–2012. Panel A: antigenemic Lassa fever cases (Ag+/IgM±). Panel B: Patients with serum anti-LASV IgM (Ag−/IgM+). Panel C: Patients with no Lassa virus seropositivity (Ag−/IgM−). The monthly frequency distributions differed between each of the serostatus group comparisons as assessed using a Poisson regression model (p<.001 for all serostatus comparisons; data not shown).

Studies conducted prior to the end of hostilities suggested that the highest incidence of LF in Sierra Leone occurs during the dry season (November to April) [1], [28]. We found a similar pattern in the post-conflict period, with a peak of antigenemic LF cases presenting to the KGH Lassa Ward in March (Fig. 5A). Ag−/IgM+ patients presented with a peak in the dry season (March) and another peak in the rainy season (October; Fig. 5B). Patients with NLFI had a distinct peak of presentation in June and July, which begins the period of greatest rainfall (Fig. 5C).

### Age distribution of suspected LF cases

**Figure 6:**
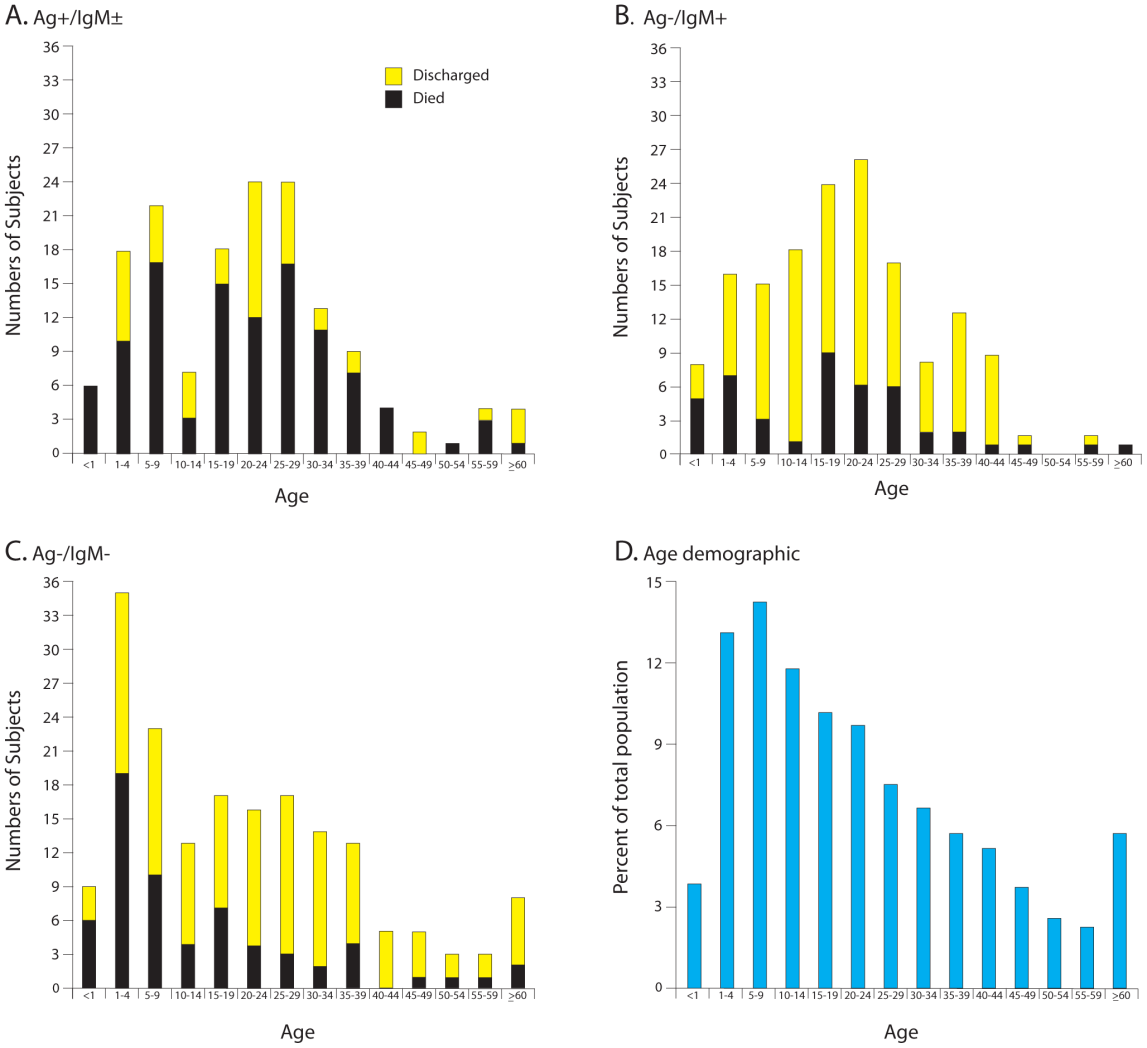
Age distribution of cases presenting to the KGH Lassa Ward, 2008–12. Panel A: Age distributions of patients presenting while antigenemic (Ag+/IgM±). Panel B: Age distributions of nonantigenemic patients presenting with serum anti-LASV IgM (Ag−/IgM+). Panel C: Age distributions of nonantigenemic patients presenting without anti-LASV IgM seropositivity (Ag−/IgM−). In Panels A–C yellow portion of bars represent patients who were discharged and black portion of bars represent patients who died. Panel D: Age demographic for the population of Sierra Leone (2010 estimate). Among patients who died, the age distributions differed significantly between the Ag+/IgM± and Ag−/IgM− groups (p = .005). Distributional comparisons were carried out using the Kolmogorov-Smirnov technique (Table S5).

Presentation of Ag+/IgM± patients occurred with a bimodal frequency, with peak incidences in childhood (ages 0–9) and in adolescence/adulthood (ages 15–39) (Fig. 6A, Table S5). In contrast, the overall age distribution observed in Ag−/IgM+ patients showed peaked in adolescence/adulthood (ages 15–39) (Fig. 6B). The age distribution of persons presenting with a NLFI (Ag−/IgM−) peaked in infancy and declined with increasing age (Fig. 6C) and was similar to the age distribution of the general Sierra Leonean population (Fig. 6D). High CFRs were observed in LASV antigenemic patients across all age groups (Fig. 6A). All antigenemic infants under the age of 1 died. Deaths in antigenemic patients (Ag+/IgM±) were bimodal, peaking in early childhood (ages 0–9) and in adolescence/early adulthood (ages 15–29; Fig. 6A) following the pattern of overall presentation. A similar bimodal distribution of patient deaths was observed in Ag−/IgM+ patients (Fig. 6B). It is unclear whether there is enhanced immunological susceptibility to severe LASV infection at certain ages or higher exposure to the rodent reservoir. The numbers of deaths in patients presenting with a NLFI (Ag−/IgM−) were biased toward infants and children (Fig. 6C), but otherwise closely resembled the age distribution of Sierra Leone's population (Fig. 6D). The age distributions overall and for patients who died or were discharged for each serogroup are replotted as fitted curves in Supplemental Fig. S1.

### Female gender and self-reported pregnancy are risk factors in LF

**Figure 7:**
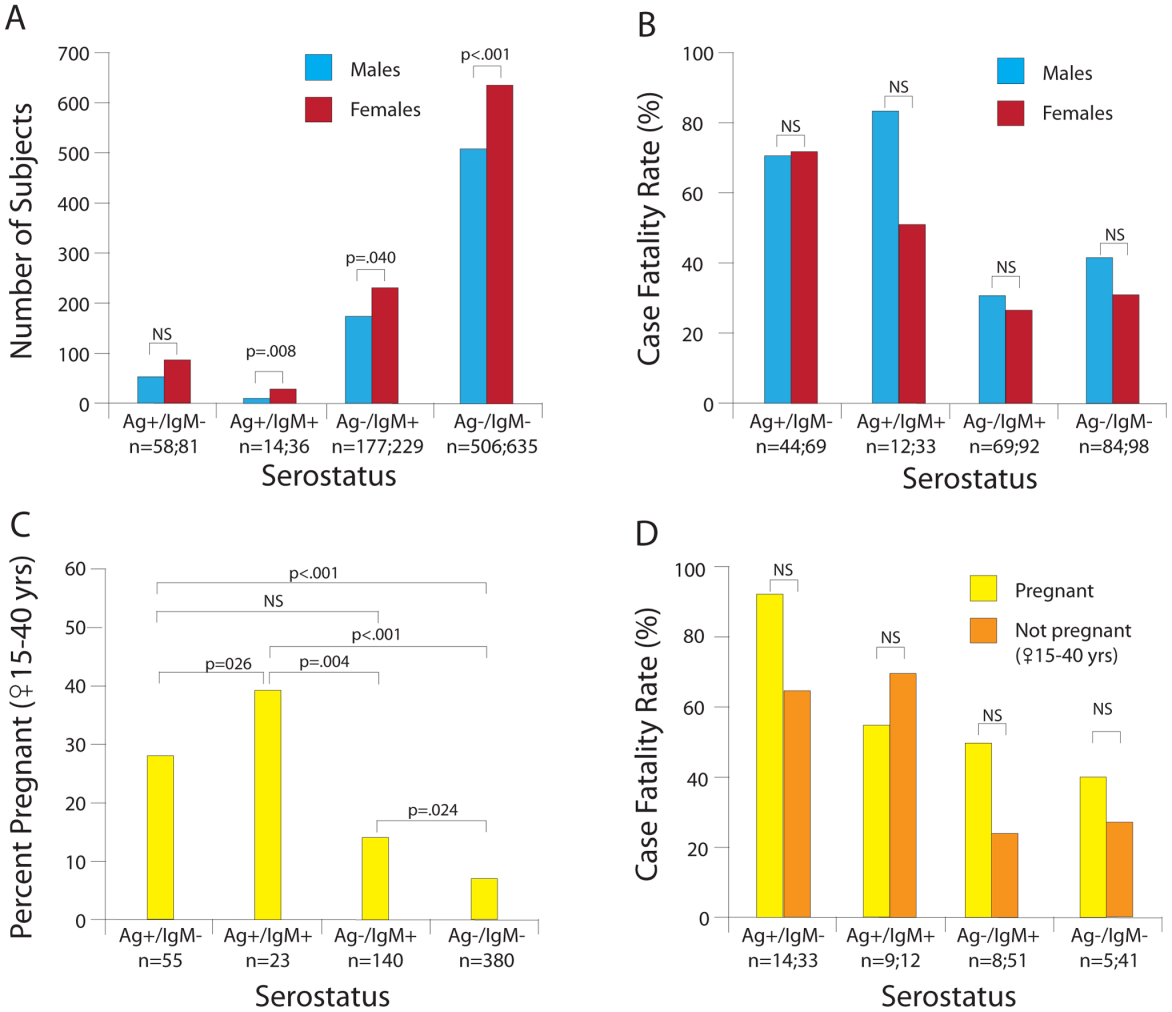
Gender and self-reported pregnancy status of suspected Lassa fever cases presenting to the KGH Lassa Ward, 2008–2012. Panel A: Frequency of suspected Lassa fever cases by gender and serostatus. Panel B: Cases fatality rates by gender and serostatus. Panel C: Percentage of female patients of childbearing age with self-reported pregnancy status by serostatus. Panel D: Case fatality rates in female patients with self-reported pregnancy status Pregnancies are self-reported and therefore likely underestimated as pregnancy tests were not routinely available. Logistic regression was used for group comparisons (Tables S6 and S7). NS = not significant.

Significantly more females than males presented to the KGH Lassa Ward with suspected LF in each serogroup, except for early-stage LF (Ag+/IgM−) (Fig. 7A). Women and men in all serogroups displayed similar CFRs (Fig. 7B). Prior studies indicated that LF in pregnancy results in a high fetal mortality and increased fatalities in pregnant woman, particularly in the last trimester [22], [29]. Women who self-reported as pregnant were significantly overrepresented in the serogroups with LASV antigenemia (Ag+/IgM−, Ag+/IgM+) compared to the Ag− serogroups (Ag−/IgM+, Ag−/IgM−; Fig. 7C). It is unlikely that infection with LASV would inform the subject regarding her pregnancy status, and first trimester pregnancies are likely underreported. We did not find a statistically significant increase in CFR related to pregnancy, as has been noted in previous studies [22], [29]. Nevertheless, the CFR for Ag+/IgM− pregnant women in our study was 93%, compared with 66% of non-pregnant women in this serogroup (Fig. 7D). The small sample size and elevated mortality noted for all groups in our study may account for the lack of statistical significance.

### Case fatality rates in ribavirin-treated and untreated patients

**Figure 8:**
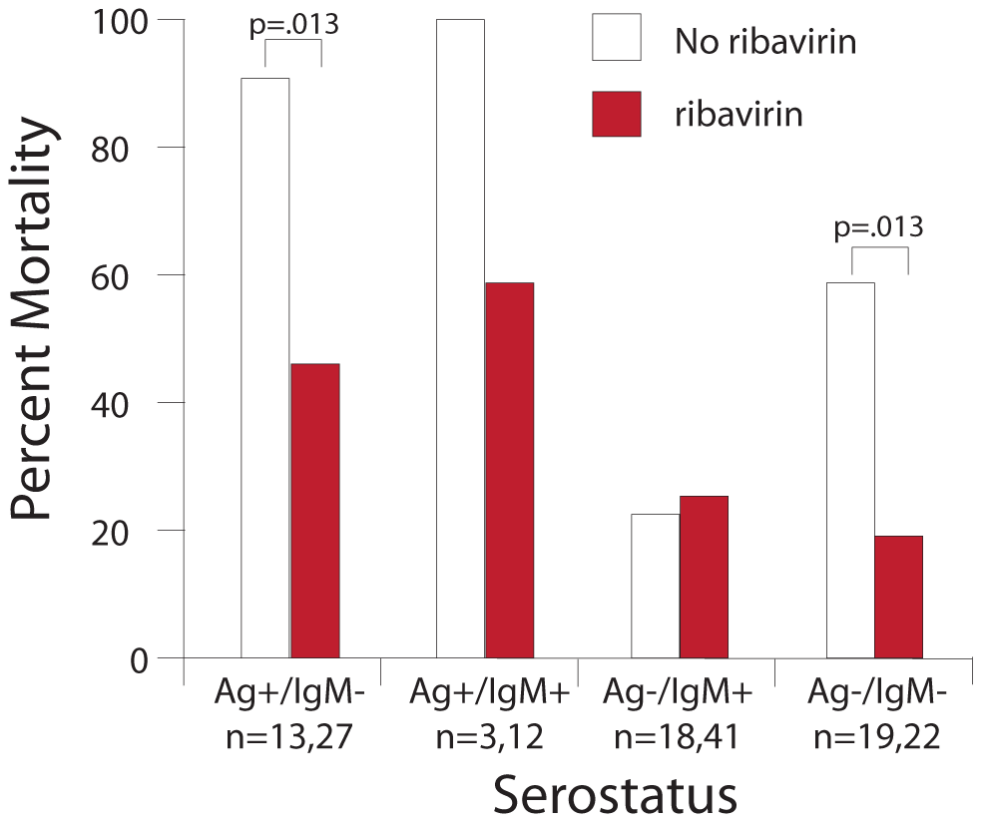
Case fatality rates for suspected LF cases by ribavirin treatment status and serostatus. The presence of LASV Ag in serum of patients with observed survival outcomes and verified treatment status was assessed by recombinant Ag− and IgM-capture ELISA. Statistical significance for within and between group comparisons was determined using a multivariate logistic regression model (Table S9). NS = not significant.

The antiviral drug ribavirin has been reported to significantly reduce LF CFRs [18]. We observed that the CFR in Ag+/IgM− patients treated with ribavirin was 44% compared to 92% in untreated subjects (Fig. 8). Fewer ribavirin-treated Ag+/IgM+ patients died than untreated patients, but the number of patients in this group was small and this difference was not statistically significant. Most Ag+ subjects whether treated or not showed a decline in LASV antigenemia; among 69 antigenemic subjects with a second blood draw (generally 1–2 days later), only 10 showed any increase in antigen load, with 16/69 with a similar load on the second blood draw and most (43/69) with a decreased load. Of these 43 patients, 29 had greater than a 10-fold reduction in virus antigen on the second blood draw. There was no difference in survival between ribavirin-treated and untreated Ag−/IgM+ patients. Unexpectedly, mortality in persons with NLFI (Ag−/IgM−) was lower in ribavirin-treated versus untreated patients.

## Discussion

The civil war that ravaged Sierra Leone from 1991 to 2002 displaced hundreds of thousands of people, destroyed health and education facilities and disrupted the training of a generation of healthcare workers. Many skilled health and research professionals fled the country. A decade later, Sierra Leone is still struggling to rebuild its health sector, but improvement in many areas is being realized. The results reported here highlight significant progress toward one of Sierra Leone's major public health goals – effective management of the LF threat. A leading LF clinical and research center has been reestablished at KGH. Numbers of suspected LF cases presenting at KGH have increased dramatically with the end of hostilities, which can be attributed, in part, to the efforts of the KGH LF outreach team, who have sensitized healthcare providers in rural health centers to the LF threat and alerted them to the renewed availability of LF testing and treatment at KGH. The increased ability to safely move about the country has likely also increased the ability of persons with LF to present for care, but may have lead to transmission to new areas.

The observed CFR in LASV antigenemic patients presenting to KGH during the period from 2008–12 was 69%. Studies published in the pre-conflict period reported overall CFRs in similar populations of LF patients of 8 to 38% [1], [6], [14], [16]. Differences in sensitivity of the different assays used to detect LASV infection coupled with alternative paradigms for classifying LF patients may account for the differences in CFRs. RT-PCR and virus culture are capable of detecting lower levels of LASV than immunoassays [14], [15], [27]. Furthermore, subjects presenting without viremia, but with anti-LASV IgM, were included in the diagnosis of acute LF in previous studies. [27], [28]. It is important to note that these prior studies used different assays than those employed in the current studies. For KGH patients presenting with anti-LASV IgM from 2008–12 the CFR was 29%. The combined CFR of Ag+ plus IgM+ patients presenting to KGH was 49%, which is still higher than observed in most studies prior to the civil conflict. In contrast to other viral infections in which antiviral IgM antibodies are replaced with predominantly IgG antibodies, anti-LASV IgM antibodies, particularly to the LASV glycoprotein and Z protein, often persist for months to years after LASV exposure [23]. Studies in non human primate also indicated that anti-LASV IgM antibodies persist [30], leading us to speculate that LASV infection dysregulates antibody class-switching [23]. These studies suggest that not all febrile patients presenting with anti-LASV IgM in the absence of antigenemia have recent LASV infection, and that including all IgM+ patients as acute LF underestimates the CFR of this disease.

It is worth mentioning that CFRs in patients presenting to health care facilities represent only the most severe LASV infections. Our hospital-based studies were not designed to determine overall exposure rates to LASV. Moreover, individuals that develop less severe manifestations of the infection or that remain asymptomatic may not seek medical care. As discussed by others previously [28], [31], [32], hospital-based CFRs do not reflect overall mortality rates caused by LASV infection, which are likely to be considerably lower.

To guide clinical management of patients with LF, it is important to monitor follow-up samples for changes in levels of anti-LASV antibodies. A rise in IgM titers or IgM to IgG seroconversion in a nonantigenemic patient indicates a post-acute stage of LF. Results of our recent clinical trial of the LF diagnostic assay system revealed that patients with changing IgM or IgG levels represent a minority of IgM+ patients presenting with febrile illnesses to the KGH Lassa Ward overall (Table S2, unpublished data). Suspected cases presenting with sustained anti-LASV IgM levels in the absence of LASV antigenemia likely have febrile illnesses other than LF. We found that LF screening with the ReLASV diagnostic assays was capable of identifying great than 95% of active LF cases (acute and post-acute) as confirmed by RT-PCR (Table S2).

While the majority of LF cases presenting to KGH were from the long-recognized endemic Kenema and Bo Districts, cases were also confirmed in areas of Sierra Leone that had not previously been considered to have a high risk for LF. We observed LF cases from ten of Sierra Leone's thirteen districts, with numerous cases from outside the traditional endemic zone. These results suggest that LASV may be more broadly distributed across Sierra Leone than was observed prior to the civil conflict. Alternatively, increased awareness due to outreach efforts and greater access for testing/transport of suspected patients to Kenema may account for the increased numbers of cases reporting from these new regions. It is also possible that LASV-infected *Mastomys natalensis* populations are expanding to new areas. We also cannot exclude the possibility that Ag+ or IgM+ persons from other regions were exposed to LASV while previously living in or transiting through the classically recognized endemic areas or from contact with infected visitors, especially considering the massive human migrations that have occurred in post-war Sierra Leone.

The highest incidence of LF in Sierra Leone occurs during the dry season (November to April), a pattern also observed prior to the conflict [31]. Our studies also confirmed reports conducted prior to the civil conflict by indicating that infants, children, young adults, and pregnant women are disproportionately impacted by LF [29], [33], [34]. Deaths in patients presenting while LASV antigenemic were highest in infants and children under 9 years of age and adolescents and adults less than 29 years of age. Women who self-reported as pregnant were significantly overrepresented in serogroups with LASV antigenemia. From 2008 to 2012 the percentage of children under five and pregnant women presenting to the KGH Lassa Ward increased from approximately 7% to over 18% of children and from 0% to 20% of women, with significant increases in 2010 (Table S7). These increases are in part attributable to Sierra Leone's groundbreaking free healthcare program for pregnant and lactating women and children under five years of age [35]. Despite these advances, Sierra Leone is still faced with high maternal, infant, and child mortality rates which may in part be attributable to LF, especially considering that LASV infection in persons with naïve or altered immune status, such as in childhood and pregnancy, appears to lead to more severe disease.

The CFR in Ag+/IgM− patients treated with ribavirin was lower than in untreated subjects (44% vs. 92%). However, these studies were not designed as a clinical trial and therefore do not permit conclusions as to the efficacy of ribavirin treatment in LF. Furthermore, the number of fatalities in treated patients was still unacceptably high, a finding largely attributable to delayed initiation of treatment [18]. From 2008–12, fewer than 25% patients with LF presented within 7 days post-symptom onset. Our lateral flow immunoassay for LASV antigen may play a role in rectifying this situation since it can be disseminated to outlying clinics and laboratories and reliably and safely performed by persons with limited laboratory training. The high CFR in ribavirin-treated subjects observed in the current studies underscores the need to develop more effective or supplemental treatments for LF. Controlled clinical studies of ribavirin efficacy are also needed, which can serve as a prelude to clinical trials of other LF treatments, including immunotherapeutics, currently in the development pipeline. Lastly, we speculate that the unexpected decrease in CFR in NLFI patients treated with ribavirin may indicate a response of unrecognized non-LASV viral infections to this drug, which has broad-spectrum antiviral properties [36]. The enhanced laboratory diagnostic capacity may serve to elucidate the pathogens inducing NLFI in patients presenting to the KGH Lassa Ward.

## Supporting Information

Figure S1 **Kernel-smoothed age distributions of suspected LF cases presenting to the KGH Lassa Ward, by serostatus group, 2008–12: An alternative presentation of** **Fig. 6** **.** Panel A: Smoothed age distributions for patients presenting while antigenemic (Ag+/IgM±, green line), patients presenting with serum anti-LASV IgM (Ag−/IgM+, red line), or no LASV seropositivity (Ag−/IgM−, blue line). Panel B: Smoothed age distributions for patients that died by serostatus. Panel C: Smoothed age distributions among patients who were discharged from the KGH by serostatus. Dotted line in panels A–C is age demographic for the population of Sierra Leone (2010 estimate). Among patients who died, the age distributions differed significantly between the Ag+/IgM+− and Ag−/IgM− groups (p = .005). A Gaussian kernel smoothing technique was used to generate the smoothed distribution curves. The distributional comparisons were carried out using the Kolmogorov-Smirnov technique (Table S5).
(TIF)

Table S1 **(corresponds to** **Fig. 1** **): Characteristics of study subjects.** This table provides characteristics of study subjects by outcome, admission status, district, age, gender pregnancy status and duration of illness.
(DOC)

Table S2 **Performance of the Recombinant Lassa Diagnostics versus RT-PCR in a clinical study conducted at KGH in 2012–13.** This table provides a summary of the performance of the immunoassays used in to evaluate the serostatus of suspected LF patients presenting to KGH.
(DOC)

Table S3 **(corresponds to** **Fig. 2** **): Logistic regression results showing serostatus case fatality ratios.** This table provides confidence intervals and p values for the data presented in Figure 2.
(DOC)

Table S4 **(corresponds to** **Fig. 3** **): Logistic regression results showing IgG-positivity ratios and serostatus case fatality ratios by IgG status.** This table provides confidence intervals and p values for the data presented in Figure 3.
(DOC)

Table S5 **(corresponds to** **Fig. 6** **and Supplemental Fig. S1): Comparison of age distributions among serostatus groups by survival outcome.** This table provides p values for the data presented in Figure 6 and Supplemental Fig. S1.
(DOC)

Table S6 **(corresponds to** **Figs. 7a and 7b** **): Logistic regression results showing gender ratios and serostatus case fatality ratios by gender.** This table provides confidence intervals and p values for the data presented in Figure 7a and 7b.
(DOC)

Table S7 **(corresponds to** **Figs. 7c and 7d** **): Logistic regression results showing pregnancy ratios and serostatus case fatality ratios by self-reported pregnancy status.** This table provides confidence intervals and p values for the data presented in Figure 7c and 7d.
(DOC)

Table S8 **Selected characteristics by year of presentation.** This table provides p values for differences in numbers of patients by gender, pregnancy status and age by year of presentation.
(DOC)

Table S9 **(corresponds to** **Figure 8** **): Logistic regression results showing serostatus case fatality ratios by treatment status.** This table provides confidence intervals and p values for the data presented in Figure 8.
(DOC)
