# Supplementary figures and images for: Lassa Fever in Post-Conflict Sierra Leone
Source: PLoS Negl Trop Dis. 2014 Mar 20;8(3):e2748. doi: 10.1371/journal.pntd.0002748 (PMC3961205; doi:10.1371/journal.pntd.0002748)

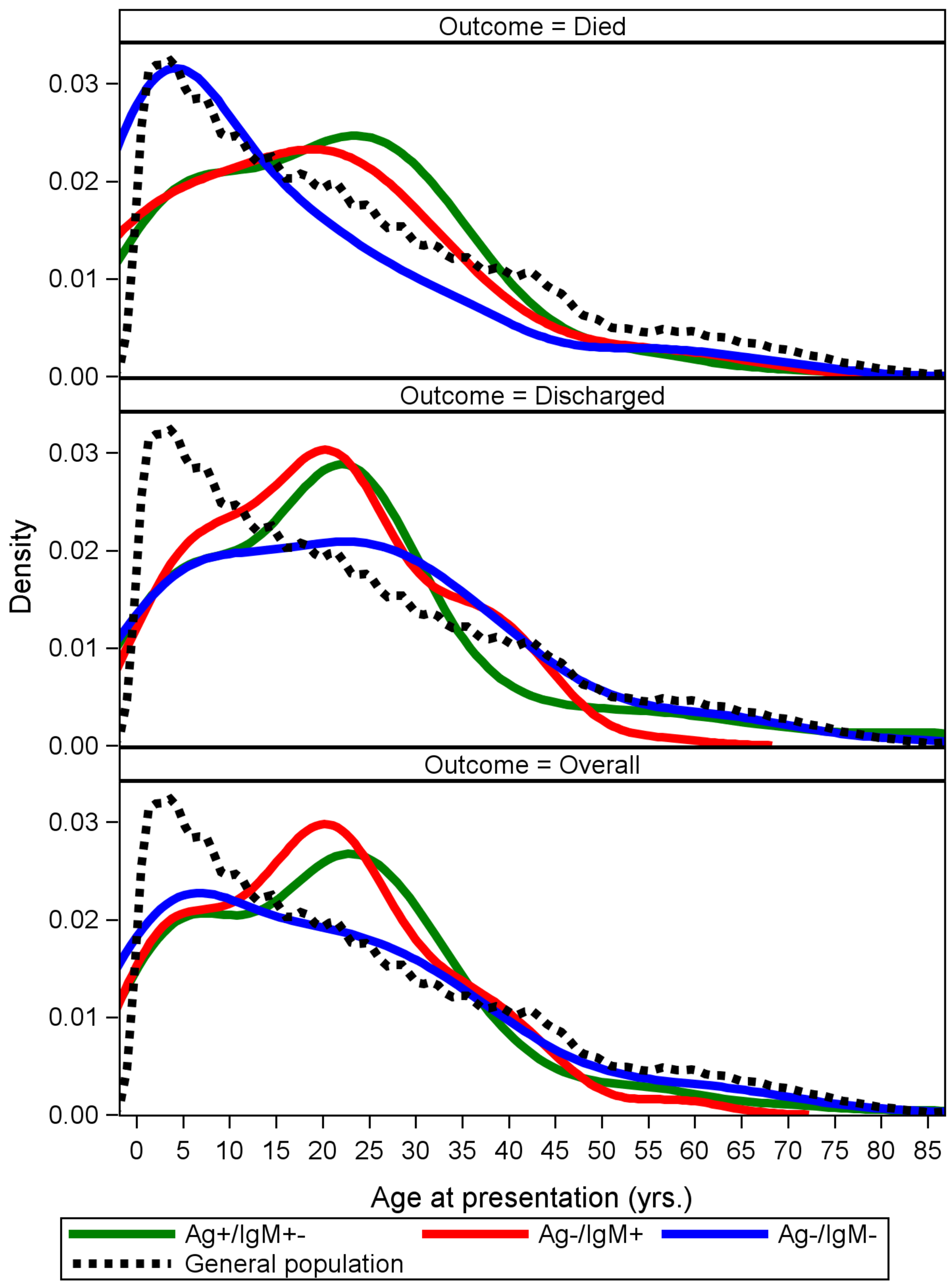

Supplement: Figure S1 — Kernel-smoothed age distributions of suspected LF cases presenting to the KGH Lassa Ward, by serostatus group, 2008–12: An alternative presentation of Fig. 6 . Panel A: Smoothed age distributions for patients presenting while antigenemic (Ag+/IgM±, green line), patients presenting with serum anti-LASV IgM (Ag−/IgM+, red line), or no LASV seropositivity (Ag−/IgM−, blue line). Panel B: Smoothed age distributions for patients that died by serostatus. Panel C: Smoothed age distributions among patients who were discharged from the KGH by serostatus. Dotted line in panels A–C is age demographic for the population of Sierra Leone (2010 estimate). Among patients who died, the age distributions differed significantly between the Ag+/IgM+− and Ag−/IgM− groups (p = .005). A Gaussian kernel smoothing technique was used to generate the smoothed distribution curves. The distributional comparisons were carried out using the Kolmogorov-Smirnov technique (Table S5). (TIF) [file pntd.0002748.s001.tif]
