# Supplementary material for: Lassa Fever in Post-Conflict Sierra Leone
Source: PLoS Negl Trop Dis. 2014 Mar 20;8(3):e2748. doi: 10.1371/journal.pntd.0002748 (PMC3961205; doi:10.1371/journal.pntd.0002748)
Supplement: Table S1 — (corresponds to Fig. 1 ): Characteristics of study subjects. This table provides characteristics of study subjects by outcome, admission status, district, age, gender pregnancy status and duration of illness. (DOC) [file pntd.0002748.s002.doc]

**Table S1. Characteristics of study subjects (corresponds to Fig. 1)**

| **Characteristic** | **Ag+/IgM-(n = 140)** | **Ag+/IgM+**  **(n = 50)** | **Ag-/IgM+**  **(n = 407)** | **Ag-/IgM-**  **(n = 1143)** | ***p*^a^** |
| --- | --- | --- | --- | --- | --- |
| **Admission status^b^** |  |  |  |  |  |
| Admitted | 87 (62) | 38 (76) | 152 (37) | 199 (17) | <.001 |
| Not admitted^c^ | 53 (38) | 12 (24) | 254 (63) | 943 (83) |  |
| **Patient outcome** |  |  |  |  |  |
| Discharged | 32 (23) | 17 (34) | 115 (28) | 117 (10) | <.001 |
| Died | 81 (58) | 28 (56) | 46 (11) | 65 (6) |  |
| Unknown^d^ | 27 (19) | 5 (10) | 246 (61) | 961 (84) |  |
|  |  |  |  |  |  |
| **District** |  |  |  |  |  |
| Kenema | 101 (72) | 34 (68) | 256 (63) | 810 (71) | .709 |
| Bo | 15 (11) | 4 (8) | 43 (10) | 123 (11) |  |
| Other in Sierra Leone | 17 (12) | 9 (18) | 40 (10) | 115 (10) |  |
| Unknown | 7 (5) | 3 (6) | 68 (17) | 95 (8) |  |
|  |  |  |  |  |  |
| **Age** |  |  |  |  |  |
| < 1 yr. | 7 (5) | 1 (2) | 12 (3) | 50 (4) | .020 |
| 1-4 yrs. | 18 (13) | 3 (6) | 49 (12) | 148 (13) |  |
| 5-14 yrs. | 23 (16) | 14 (28) | 66 (16) | 154 (14) |  |
| 15 – 40 yrs. | 77 (55) | 29 (58) | 233 (57) | 602 (53) |  |
| > 40 yrs. | 12 (9) | 3 (6) | 40 (10) | 173 (15) |  |
| Unknown | 3 (2) | 0 (0) | 7 (2) | 16 (1) |  |
| **Gender^e^** |  |  |  |  |  |
| Female | 81 (58) | 36 (72) | 229 (56) | 635 (56) | .141 |
| Male | 58 (42) | 14 (28) | 177 (44) | 506 (44) |  |
| **Pregnant^f^** |  |  |  |  |  |
| Yes | 15 (27) | 9 (39) | 19 (14) | 27 (7) | <.001 |
| No or no response | 40 (73) | 14 (61) | 121 (86) | 353 (93) |  |
| **Duration of illness^g^** |  |  |  |  |  |
| < 7 days | 26 (19) | 9 (18) | 121 (30) | 469 (41) | <.001 |
| ≥ 7 days | 83 (59) | 32 (64) | 187 (46) | 441 (39) |  |
| Unknown | 31 (22) | 9 (18) | 99 (24) | 233 (20) |  |

*Note*. All results based expressed as frequency (%) unless indicated otherwise.

^a^Corresponds to Fisher’s exact test for assessing characteristic differences among the serostatus groups; unknown classifications considered as missing for computation purposes.

^b^Admission status unknown for one Ag-/IgM+ subject and one Ag-/IgM- subject.

^c^Includes subjects dying prior to arrival at the KGH and subjects not physically presenting to the KGH (most commonly due to submitting only blood samples from a KGH referral center).

^d^Missing survival outcomes most commonly due to non-admission status.

^e^Gender unknown for one Ag+/IgM- observation, one Ag-/IgM+ observation, and two Ag-/IgM- observations.

^f^Restricted to females aged between 15 and 40 years.

^g^Defined as the number of days between dates of illness onset and initial clinical evaluation.
