# Supplementary material for: Lassa Fever in Post-Conflict Sierra Leone
Source: PLoS Negl Trop Dis. 2014 Mar 20;8(3):e2748. doi: 10.1371/journal.pntd.0002748 (PMC3961205; doi:10.1371/journal.pntd.0002748)
Supplement: Table S2 — Performance of the Recombinant Lassa Diagnostics versus RT-PCR in a clinical study conducted at KGH in 2012–13. This table provides a summary of the performance of the immunoassays used in to evaluate the serostatus of suspected LF patients presenting to KGH. (DOC) [file pntd.0002748.s003.doc]

| **Table S2. Performance of the Recombinant Lassa Diagnostics versus RT-PCR in a clinical study conducted at KGH in 2012-13.*** | | | | | | | |
| --- | --- | --- | --- | --- | --- | --- | --- |
|  | Sensitivity  (95% CI) | Specificity  (95% CI) | Positive Predictive Value  (95% CI) | Negative Predictive Value  (95% CI) | Odds Ratio  (95% CI) | Diagnostic Likelihood Ratio | Case Fatality Rate (%) |
| ReLASV Ag ELISA^1^ | 95.5  (77.2 – 99.9) | 97.8  (92.3 – 99.7) | 91.3  (72.0 – 98.9) | 98.9  (94.0 – 100) | 935  (81 – 10,804) | 43.4 | 69.6 |
| ReLASV Ag RDT^1^ | 81.2  (59.7 – 94.8) | 99.1  (95.3 – 100) | 94.7  (74.0 – 99.9) | 84.9  (78.2 – 90.2) | 513  (54.2 – 4844) | 54.9 | 70.0 |
| ReLASV IgM ELISA^2^ | 86.4  (65.1 – 97.1) | 77.6  (70.9 - 83.4) | 31.7  (20.3 - 45.0) | 97.9  (94.1 - 99.6) | 21.9  (6.2 - 77.8) | 3.85 | 6.6 |
| ReLASV IgG ELISA^2^ | 40.9  (20.7 – 63.6) | 74.3  (67.4 – 80.5) | 16.1  (7.6 - 28.3) | 91.3  (85.5 - 95.3) | 2.0  (0.8 - 5.0) | 1.59 | 7.1 |
| ^1^ First draw testing of suspected LF cases which were seronegative for LASV specific IgM or IgG based on ELISA results.  ^2^ First draw testing of suspected LF cases which were negative for LASV NP antigenemia based on Ag RDT or ELISA results.  *Enrollment Criteria for the clinical study was based on the suspected LF case definition (Table 1) [19]. LF Case Classifications: Confirmed LF: Ag±, IgM ± with positive RT-PCR* N = 47 **or** Ag-, PCR- with increasing IgM N=2 **or** Ag-, PCR- with observed IgM to IgG class-switch N=8; Non-LF: Ag-, IgM ±, IgG± with negative RT-PCR (no IgM to IgG class-switch observed) N=188; SL Controls: Asymptomatic Contacts with Non-LF diagnostic profile N=89. LASV RT-PCR was performed as described in by Olschlager and coworkers [26]. LF screening with the ReLASV diagnostic assays, upon presentation to the LF clinical study program, was capable of identifying 95% of active LF cases as confirmed by RT-PCR, rising IgM titers or IgM to IgG seroconversion. A manuscript describing the results of the clinical trial is in preparation. | | | | | | | |
