# Supplementary material for: Lassa Fever in Post-Conflict Sierra Leone
Source: PLoS Negl Trop Dis. 2014 Mar 20;8(3):e2748. doi: 10.1371/journal.pntd.0002748 (PMC3961205; doi:10.1371/journal.pntd.0002748)
Supplement: Table S3 — (corresponds to Fig. 2 ): Logistic regression results showing serostatus case fatality ratios. This table provides confidence intervals and p values for the data presented in Figure 2. (DOC) [file pntd.0002748.s004.doc]

**Table S3. Logistic regression results showing case fatality ratios for pairwise serostatus combinations (corresponds to Fig. 2)**

| **Corresponding figure** | **Comparison** | **OR (95% CI)^a^** | ***p*** |
| --- | --- | --- | --- |
| 2a | Ag+/IgM- vs. Ag+/IgM+ | 1.5 (0.7, 3.2) | .248 |
|  | vs. Ag-/IgM+ | 6.3 (3.7, 10.8) | <.001 |
|  | vs. Ag-/IgM- | 4.6 (2.7, 7.6) | <.001 |
|  | Ag+/IgM+ vs. Ag-/IgM+ | 4.1 (2.1, 8.2) | <.001 |
|  | vs. Ag-/IgM- | 3.0 (1.5, 5.8) | .002 |
|  | Ag-/IgM+ vs. Ag-/IgM- | 0.7 (0.5, 1.1) | .159 |
| 2b | Ag+/IgM+- or Ag-/IgM+ vs. Ag-/IgM- | 1.7 (1.2, 2.5) | .005 |

*Note*. OR = odds ratio; CI = confidence interval. ^a^Odds ratios expressed as the odds of a fatal outcome vs. the reference group.
