# Supplementary material for: Lassa Fever in Post-Conflict Sierra Leone
Source: PLoS Negl Trop Dis. 2014 Mar 20;8(3):e2748. doi: 10.1371/journal.pntd.0002748 (PMC3961205; doi:10.1371/journal.pntd.0002748)
Supplement: Table S4 — (corresponds to Fig. 3 ): Logistic regression results showing IgG-positivity ratios and serostatus case fatality ratios by IgG status. This table provides confidence intervals and p values for the data presented in Figure 3. (DOC) [file pntd.0002748.s005.doc]

**Table S4. Logistic regression results showing IgG-positivity ratios and case fatality ratios by IgG status (corresponds to Figs. 3a and 3b)**

| **Corresponding figure** | **Comparison, stratum** | **OR (95% CI)** | ***p*** |
| --- | --- | --- | --- |
| 3a^a^ | Ag+/IgM- vs. Ag+/IgM+ | 0.4 (0.1, 1.3) | .136 |
|  | vs. Ag-/IgM+ | 0.1 (0.1, 0.3) | <.001 |
|  | vs. Ag-/IgM- | 0.4 (0.2, 0.8) | .014 |
|  | Ag+/IgM+ vs. Ag-/IgM+ | 0.3 (0.1, 0.8) | .017 |
|  | vs. Ag-/IgM- | 0.9 (0.4, 2.2) | .818 |
|  | Ag-/IgM+ vs. Ag-/IgM- | 2.7 (2.0, 3.6) | <.001 |
| 3b^b^ | Ag+/IgM- vs. Ag+/IgM+, IgG+ | 0.1 (0.0, 2.0) | .141 |
|  | vs. Ag-/IgM+, IgG+ | 1.0 (0.2, 6.3) | .970 |
|  | vs. Ag-/IgM-, IgG+ | 2.7 (0.3, 26.6) | .383 |
|  | Ag+/IgM+ vs. Ag-/IgM+, IgG+ | 8.3 (0.8, 81.2) | .069 |
|  | vs. Ag-/IgM-, IgG+ | 22.0 (1.5, 314.2) | .023 |
|  | Ag-/IgM+ vs. Ag-/IgM-, IgG+ | 2.7 (0.5, 13.6) | .242 |
|  | Ag+/IgM- vs. Ag+/IgM+, IgG- | 2.0 (0.8, 5.1) | .160 |
|  | vs. Ag-/IgM+, IgG- | 6.8 (3.3, 14.1) | <.001 |
|  | vs. Ag-/IgM-, IgG- | 3.4 (1.6, 7.2) | .001 |
|  | Ag+/IgM+ vs. Ag-/IgM+, IgG- | 3.4 (1.3, 8.7) | .010 |
|  | vs. Ag-/IgM-, IgG- | 1.7 (0.7, 4.4) | .263 |
|  | Ag-/IgM+ vs. Ag-/IgM-, IgG- | 0.5 (0.2, 1.0) | .064 |
|  | IgG+ vs. IgG-, Ag+/IgM- | 0.2 (0.0, 0.9) | .042 |
|  | IgG+ vs. IgG-, Ag+/IgM+ | 2.5 (0.2, 25.7) | .441 |
|  | IgG+ vs. IgG-, Ag-/IgM+ | 1.0 (0.5, 2.3) | .941 |
|  | IgG+ vs. IgG-, Ag-/IgM- | 0.2 (0.0, 1.0) | .045 |
| 3c^b^ | Acute vs. Convalescent | 5.0 (2.3, 10.6) | <.001 |
|  | Acute vs. Non-LF | 4.1 (2.4, 7.0) | <.001 |
|  | Convalescent vs. Non-LF | 0.8 (0.4, 1.7) | .600 |

*Note*. OR = odds ratio; CI = confidence interval.

^a^Corresponding ORs expressed as the odds of IgG-positivity relative to the reference group. ^b^Corresponding ORs expressed as the odds of a fatal survival outcome relative to the reference group.
