# Supplementary material for: Lassa Fever in Post-Conflict Sierra Leone
Source: PLoS Negl Trop Dis. 2014 Mar 20;8(3):e2748. doi: 10.1371/journal.pntd.0002748 (PMC3961205; doi:10.1371/journal.pntd.0002748)
Supplement: Table S5 — (corresponds to Fig. 6 and Supplemental Fig. S1): Comparison of age distributions among serostatus groups by survival outcome. This table provides p values for the data presented in Figure 6 and Supplemental Fig. S1. (DOC) [file pntd.0002748.s006.doc]

**Table S5. Comparison of age distributions for serostatus groups by survival outcome (corresponds to Fig. 6).**

| **Outcome** | **Comparison** | ***p^a^*** |
| --- | --- | --- |
| Died | Ag+/IgM+- vs. Ag-/IgM+ | .354 |
|  | vs. Ag-/IgM- | .007 |
|  | Ag-/IgM+ vs. Ag-/IgM- | .066 |
| Discharged | Ag+/IgM+- vs. Ag-/IgM+ | .845 |
|  | vs. Ag-/IgM- | .528 |
|  | Ag-/IgM+ vs. Ag-/IgM- | .299 |

^a^Determined using the Kolmogorov-Smirnov test for distributional comparisons.
