# Supplementary material for: Lassa Fever in Post-Conflict Sierra Leone
Source: PLoS Negl Trop Dis. 2014 Mar 20;8(3):e2748. doi: 10.1371/journal.pntd.0002748 (PMC3961205; doi:10.1371/journal.pntd.0002748)
Supplement: Table S6 — (corresponds to Figs. 7a and 7b ): Logistic regression results showing gender ratios and serostatus case fatality ratios by gender. This table provides confidence intervals and p values for the data presented in Figure 7a and 7b. (DOC) [file pntd.0002748.s007.doc]

**Table S6. Logistic regression results showing gender ratios and case fatality ratios by gender (corresponds to Figs. 7a and 7b)**

| **Corresponding figure** | **Comparison** | **OR (95% CI)** | ***p*** |
| --- | --- | --- | --- |
| 7a^a^ | Ag+/IgM- vs. Ag+/IgM+ | 0.5 (0.3, 1.1) | .089 |
|  | vs. Ag-/IgM+ | 1.1 (0.7, 1.6) | .701 |
|  | vs. Ag-/IgM- | 1.1 (0.8, 1.6) | .557 |
|  | Ag+/IgM+ vs. Ag-/IgM+ | 2.0 (1.0, 3.8) | .038 |
|  | vs. Ag-/IgM- | 2.0 (1.1, 3.8) | .025 |
|  | Ag-/IgM+ vs. Ag-/IgM- | 1.0 (0.8, 1.3) | .794 |
| 7b^b^ | Ag+/IgM- vs. Ag+/IgM+, females | 2.1 (0.9, 5.2) | .075 |
|  | vs. Ag-/IgM+, females | 7.5 (3.7, 15.1) | <.001 |
|  | vs. Ag-/IgM-, females | 5.7 (2.9, 11.2) | <.001 |
|  | Ag+/IgM+ vs. Ag-/IgM+, females | 3.4 (1.5, 7.8) | .004 |
|  | vs. Ag-/IgM-, females | 2.6 (1.2, 5.8) | .021 |
|  | Ag-/IgM+ vs. Ag-/IgM-, females | 0.8 (0.4, 1.4) | .400 |
|  | Ag+/IgM- vs. Ag+/IgM+, males | 0.5 (0.1, 2.5) | .379 |
|  | vs. Ag-/IgM+, males | 5.1 (2.2, 11.6) | <.001 |
|  | vs. Ag-/IgM-, males | 3.5 (1.6, 7.7) | .002 |
|  | Ag+/IgM+ vs. Ag-/IgM+, males | 10.7 (2.2, 52.9) | .004 |
|  | vs. Ag-/IgM-, males | 7.4 (1.5, 35.7) | .013 |
|  | Ag-/IgM+ vs. Ag-/IgM-, males | 0.7 (0.4, 1.3) | .273 |
|  | Females vs. males, Ag+/IgM- | 1.1 (0.5, 2.5) | .817 |
|  | Females vs. males, Ag+/IgM+ | 0.2 (0.0, 1.3) | .093 |
|  | Females vs. males, Ag-/IgM+ | 0.8 (0.4, 1.5) | .421 |
|  | Females vs. males, Ag-/IgM- | 0.7 (0.4, 1.3) | .215 |

*Note*. OR = odds ratio; CI = confidence interval

^a^Associated ORs expressed as the odds of female presentation relative to the reference group. ^b^Associated ORs expressed as the odds of a fatal survival outcome relative to the reference group.
