# Supplementary material for: Lassa Fever in Post-Conflict Sierra Leone
Source: PLoS Negl Trop Dis. 2014 Mar 20;8(3):e2748. doi: 10.1371/journal.pntd.0002748 (PMC3961205; doi:10.1371/journal.pntd.0002748)
Supplement: Table S7 — (corresponds to Figs. 7c and 7d ): Logistic regression results showing pregnancy ratios and serostatus case fatality ratios by self-reported pregnancy status. This table provides confidence intervals and p values for the data presented in Figure 7c and 7d. (DOC) [file pntd.0002748.s008.doc]

**Table S7. Logistic regression results showing pregnancy ratios and case fatality**

**ratios by self-reported pregnancy status (corresponds to Figs. 7c and 7d):**

| **Corresponding figure** | **Comparison** | **OR (95% CI)** | ***p*** |
| --- | --- | --- | --- |
| 7c^a,b^ | Ag+/IgM- vs. Ag+/IgM+ | 0.6 (0.2, 1.6) | .303 |
|  | vs. Ag-/IgM+ | 2.4 (1.1, 5.1) | .026 |
|  | vs. Ag-/IgM- | 4.9 (2.4, 10.0) | <.001 |
|  | Ag+/IgM+ vs. Ag-/IgM+ | 4.1 (1.6, 10.8) | .004 |
|  | vs. Ag-/IgM- | 8.4 (3.3, 21.2) | <.001 |
|  | Ag-/IgM+ vs. Ag-/IgM- | 2.1 (1.1, 3.8) | .024 |
| 7d^a,c^ | Ag+/IgM- vs. Ag+/IgM+, pregnant | 10.4 (0.9, 117.2) | .058 |
|  | vs. Ag-/IgM+, pregnant | 13.0 (1.1, 152.3) | .041 |
|  | vs. Ag-/IgM-, pregnant | 19.5 (1.3, 292.7) | .032 |
|  | Ag+/IgM+ vs. Ag-/IgM+, pregnant | 1.3 (0.2, 8.4 ) | .819 |
|  | vs. Ag-/IgM-, pregnant | 1.9 (0.2, 17.3) | .579 |
|  | Ag-/IgM+ vs. Ag-/IgM-, pregnant | 1.5 (0.2, 14.4) | .726 |
|  | Ag+/IgM- vs. Ag+/IgM+, NP | 1.1 (0.3, 4.1) | .894 |
|  | vs. Ag-/IgM+, NP | 5.1 (2.0, 13.2) | <.001 |
|  | vs. Ag-/IgM-, NP | 5.4 (2.0, 14.8) | .001 |
|  | Ag+/IgM+ vs. Ag-/IgM+, NP | 4.7 (1.3, 16.9) | .018 |
|  | vs. Ag-/IgM-, NP | 5.0 (1.3, 18.7) | .018 |
|  | Ag-/IgM+ vs. Ag-/IgM-, NP | 1.1 (0.4, 2.7) | .904 |
|  | Pregnant vs. NP, Ag+/IgM- | 7.4 (0.9, 64.0) | .068 |
|  | Pregnant vs. NP, Ag+/IgM+ | 0.8 (0.1, 4.4) | .779 |
|  | Pregnant vs. NP, Ag-/IgM+ | 2.9 (0.6, 13.4) | .167 |
|  | Pregnant vs. NP, Ag-/IgM- | 2.1 (0.3, 14.2) | .460 |

*Note*. OR = odds ratio; CI = confidence interval; NP = nonpregnant.

^a^Sample covers females between 15 and 40 years of age. ^b^Associated ORs expressed as the odds of observed pregnancy on presentation relative to the reference group. ^c^Associated ORs expressed as the odds of a fatal outcome relative to the reference group.
