# Supplementary material for: Lassa Fever in Post-Conflict Sierra Leone
Source: PLoS Negl Trop Dis. 2014 Mar 20;8(3):e2748. doi: 10.1371/journal.pntd.0002748 (PMC3961205; doi:10.1371/journal.pntd.0002748)
Supplement: Table S8 — Selected characteristics by year of presentation. This table provides p values for differences in numbers of patients by gender, pregnancy status and age by year of presentation. (DOC) [file pntd.0002748.s009.doc]

**Table S8. Selected characteristics by year of presentation**

| **Characteristic** | **Year of presentation** | | | | |  |
| --- | --- | --- | --- | --- | --- | --- |
|  | **2008** | **2009** | **2010** | **2011** | **2012** | ***p*^a^** |
| **Gender** |  |  |  |  |  |  |
| Female | 44 (52) | 60 (58) | 236 (53) | 339 (59) | 302 (57) | .221 |
| Male | 41 (48) | 43 (42) | 210 (47) | 234 (41) | 227 (43) |  |
| **Pregnancy status^b^** |  |  |  |  |  |  |
| Pregnant | 0 (0) | 2 (5) | 10 (8) | 20 (10) | 38 (20) | <.001 |
| Nonpregnant | 28 (100) | 42 (95) | 122 (92) | 187 (90) | 149 (80) |  |
| **Age** |  |  |  |  |  |  |
| < 5 yrs. | 6 (7) | 8 (8) | 87 (20) | 95 (17) | 92 (18) | .034 |
| ≥ 5 yrs. | 79 (93) | 95 (92) | 356 (80) | 470 (83) | 426 (82) |  |

*Note*. Results are expressed as n (%) unless noted otherwise.

^a^Based on the Cochran-Armitage test for trend. ^b^Restricted to females aged between 15 and 40 yrs.=
