# Supplementary material for: Lassa Fever in Post-Conflict Sierra Leone
Source: PLoS Negl Trop Dis. 2014 Mar 20;8(3):e2748. doi: 10.1371/journal.pntd.0002748 (PMC3961205; doi:10.1371/journal.pntd.0002748)
Supplement: Table S9 — (corresponds to Figure 8 ): Logistic regression results showing serostatus case fatality ratios by treatment status. This table provides confidence intervals and p values for the data presented in Figure 8. (DOC) [file pntd.0002748.s010.doc]

**Table S9. Logistic regression results showing serostatus case fatality ratios by treatment status (corresponds to Figure 8)**

| **Comparison, stratum** | **OR (95% CI)^a^** | ***p*** |
| --- | --- | --- |
|
| Ag+/IgM- vs. Ag+/IgM+, untreated | 1.2 (0.0, 36.1) | .920 |
| vs. Ag-/IgM+, untreated | 26.9 (3.6, 197.8) | .001 |
| vs. Ag-/IgM-, untreated | 6.2 (0.9, 41.7) | .062 |
| Ag+/IgM+ vs. Ag-/IgM+, untreated | 22.6 (0.9, 524.4) | .052 |
| vs. Ag-/IgM-, untreated | 5.2 (0.2, 114.0) | .298 |
| Ag-/IgM+ vs. Ag-/IgM-, untreated | 0.2 (0.1, 0.9) | .037 |
| Ag+/IgM- vs. Ag+/IgM+, treated | 0.6 (0.2, 2.2) | .439 |
| vs. Ag-/IgM+, treated | 2.4 (0.9, 6.7) | .090 |
| vs. Ag-/IgM-, treated | 3.3 (0.9, 11.8) | .065 |
| Ag+/IgM+ vs. Ag-/IgM+, treated | 4.1 (1.1, 15.1) | .034 |
| vs. Ag-/IgM-, treated | 5.6 (1.2, 25.3) | .025 |
| Ag-/IgM+ vs. Ag-/IgM-, treated | 1.4 (0.4, 4.8) | .620 |
| Untreated vs. treated, Ag+/IgM- | 10.3 (1.6, 65.7) | .013 |
| Untreated vs. treated, Ag+/IgM+ | 5.1 (0.2, 121.1) | .310 |
| Untreated vs. treated, Ag-/IgM+ | 0.9 (0.3, 3.3) | .912 |
| Untreated vs. treated, Ag-/IgM- | 5.6 (1.4, 21.7) | .013 |

*Note*. OR = odds ratio; CI = confidence interval. ^a^Odds ratios are expressed as the odds of a fatal survival outcome relative to the reference group.
